# Predicting EGFR gene mutation in lung adenocarcinoma using spectral CT combined with AI parameters: a diagnostic accuracy study

**DOI:** 10.3389/fonc.2025.1611759

**Published:** 2025-10-30

**Authors:** Lilan She, Min Xie, Guolin Xu, Xiangmei Zhan, Meilan Huang, Yunjing Xue

**Affiliations:** Department of Radiology, Fujian Medical University Union Hospital, Fuzhou, China

**Keywords:** spectral CT, artificial intelligence, lung adenocarcinoma, EGFR mutation, predictive model

## Abstract

**Purpose:**

Epidermal growth factor receptor(EGFR) mutation is one of the most critical biomarkers in non-small cell lung cancer (NSCLC), holding significant clinical implications for guiding targeted therapy selection and prognostic assessment in patients. This study aims to evaluate the predictive value of spectral CT parameters, artificial intelligence (AI)-derived parameters, and clinical indicators for EGFR mutation in lung adenocarcinoma.

**Methods:**

This retrospective study analyzed 150 patients with pathologically confirmed lung adenocarcinoma. All patients underwent EGFR genotyping, non-contrast CT, and spectral contrast-enhanced CT. Spectral parameters included spectral curve slope (λHU), iodine concentration (IC), water concentration (WC), Effective atomic number (Effective-Z), and CT values at 70 keV. An AI-assisted diagnostic system automatically extracted quantitative AI parameters: The three-dimensional (3D) radiomic features(including long-axis diameter, short-axis diameter, surface area, 3D long-axis diameter, maximum cross-sectional area, volume), CT attenuation histogram features(including solid component percentage, mean CT value, median CT value, CT value standard deviation, maximum CT values, minimum CT values, kurtosis, skewness, energy, and entropy)and morphological characteristics(including compactness, sphericity). Correlations between spectral CT parameters, AI parameters, clinical variables, and EGFR mutation status were assessed. Independent predictors were identified via multivariate analysis to construct a predictive model.

**Results:**

Univariate analysis revealed associations between EGFR mutation and gender (P = 0.013), smoking history (P = 0.001), λHU (P = 0.049), and tumor surface area (P = 0.043). Multivariate analysis identified smoking history (P = 0.012), λHU (P = 0.015), and surface area (P = 0.029) as independent predictors. The predictive model integrating these three factors achieved an AUC of 0.713 (95% CI: 0.628–0.797), a specificity of 0.754, and a sensitivity of 0.600, demonstrating moderate diagnostic accuracy. Calibration curves indicated good agreement between predicted and observed probabilities, while decision curve analysis confirmed clinical utility.

**Conclusion:**

The integration of spectral CT and AI-derived quantitative parameters with clinical indicators demonstrates significant potential for noninvasive prediction of EGFR mutation in lung adenocarcinoma. This non-invasive predictive approach could reduce unnecessary invasive biopsies. Particularly in patients with contraindications to invasive procedures, this model offers a viable alternative.

## 1 Introduction

Lung cancer remains one of the most prevalent malignancies worldwide, ranking second in incidence and exhibiting persistently high mortality rates (1). NSCLC constitutes the predominant histological subtype, with lung adenocarcinoma being the most common form of NSCLC. EGFR mutations represent the most frequent driver genetic alterations in Asian populations (2, 3). Non-smokers exhibit a significantly higher prevalence of EGFR mutations compared to smokers, with these mutations predominantly manifesting as pure ground-glass nodules (GGNs) or part-solid nodules—particularly among Asian females. Consequently, low-dose CT (LDCT) screening is critically important in this population, as it may reflect EGFR status through discernible CT characteristics (4, 5).Tyrosine kinase inhibitors (TKIs) have demonstrated significant efficacy in suppressing. EGFR-mediated oncogenic signaling (6–8), underscoring the clinical imperative to accurately predict EGFR mutation status for guiding therapeutic decision-making. However, current methods for obtaining EGFR genotypic information predominantly rely on postoperative or biopsy specimens, which are invasive, cost-prohibitive, and poorly tolerated by certain patients. In recent years, novel EGFR detection methodologies have emerged, exemplified by liquid biopsy platforms incorporating circulating tumor cells (CTCs), circulating tumor DNA (ctDNA), and exosomes. However, their ultra-low concentrations in biofluids and suboptimal detection rates necessitate stringent technical requirements. Compared to histological biopsy, liquid biopsy demonstrates elevated false-negative rates and compromised sensitivity profiles (9–11). In contrast, predictions based on non-invasive imaging show promise, such as quantitative parameters from CT radiomics or deep learning, but they usually focus on single-modality features (12, 13).

Spectral CT, utilizing rapid voltage switching between high- and low-energy spectra (80 keV and 140 keV), provides quantitative multiparametric data, including spectral curve analysis, iodine-water decomposition maps, Effective-Z, and monoenergetic imaging. These parameters offer critical insights into tumor compositional heterogeneity. Concurrently, AI has achieved remarkable advancements in medical imaging, enabling automated extraction of high-dimensional tumor features (14). AI-based systems not only reduce radiologists’ workload but also enhance the detection accuracy of pulmonary nodules (15, 16). The integration of AI-derived quantitative parameters with spectral CT biomarkers demonstrates synergistic advantages, significantly reinforcing the theoretical foundation of predictive models. AI excels at capturing high-dimensional morphological and textural features, which reflect macroscopic tumor architecture and intratumoral heterogeneity. These parameters correlate with tumor growth patterns and aggressiveness, potentially linked to EGFR-driven oncogenic pathways. In contrast, spectral CT generates IC, λHU, and Effective-Z, providing functional insights into tumor biology. These parameters are directly influenced by EGFR-mediated angiogenesis. The complementary value of these modalities lies in their ability to address distinct aspects of tumor characteristics. Furthermore, in spectral CT analysis, where ROI delineation is inherently observer-dependent, AI-driven automation provides maximal assurance of standardized and reproducible parameter extraction. This study aims to integrate spectral CT parameters, AI-derived quantitative biomarkers, and clinical indicators to investigate their correlations with EGFR mutation status. By constructing a predictive model, we seek to establish a noninvasive approach for precise EGFR mutation profiling in lung adenocarcinoma.

## 2 Materials and methods

### 2.1 Study population

This retrospective study enrolled 150 patients with lung adenocarcinoma treated at our institution’s Department of Thoracic Surgery between January 2019 and July 2023. Inclusion criteria: (1) completion of contrast-enhanced chest CT within 1 month preoperatively or pre-biopsy; (2) pathological confirmation of lung adenocarcinoma with comprehensive genetic profiling; (3) diagnostic-quality imaging. Exclusion criteria: (1) prior tumor-modifying interventions before CT; (2) coexisting extra-thoracic malignancies; (3) EGFR mutations co-occurring with other driver mutations. Clinical variables included sex, age, smoking history, family history of lung cancer, and prior malignancies. Smoking history was defined as either current smoking (≥1 cigarette/day for >6 consecutive months) or former smoking (cessation duration >6 months). This study is a retrospective observational study approved by the hospital’s ethics committee. In accordance with the Declaration of Helsinki, informed consent was waived due to the use of existing anonymized data, the non-interference with established treatment plans, and the absence of additional risk to the participants.

### 2.2 CT acquisition protocol

All scans were performed using a Revolution CT scanner (GE Healthcare, USA). Non-contrast and spectral contrast-enhanced scans covered the entire thorax during breath-hold after deep inspiration. Standard non-contrast parameters: 120 kV tube voltage, automatic tube current modulation, pitch 0.992, rotation time 0.5 s. Spectral imaging used Gemstone Spectral Imaging (GSI) mode: 445 mA tube current, pitch 0.992, rotation time 0.5 s. All patients received intravenous administration of ioversol contrast agent (350 mgI/mL) via the antecubital vein using the following protocol: injection volume 1.0 mL/kg (60–80 mL total dose) at a rate of 2.5 mL/s. Scan initiation was delayed for 55 seconds post-injection to ensure sufficient time for contrast agent perfusion of the lesions, thereby enabling more accurate assessment of tumor vascularity.

### 2.3 Spectral image analysis

Reconstructed 1.25-mm spectral images (mediastinal window settings: width 400 HU; level 40 HU) were transferred to an AW 4.7 workstation for GSI analysis. Regions of interest (ROI) were manually delineated on the largest cross-sectional area of tumor solid components, avoiding cavitations, blood vessels, and calcifications (ROI area ≥2/3 of solid components). Quantitative parameters derived from material decomposition maps included iodine concentration (IC), water concentration (WC), and CT values at 70 keV. Spectral curve slope (λHU) was calculated as: λ_HU_=(CT40 keV-CT100 keV)/(100keV-40keV). λHU represents the rate of change in X-ray attenuation between the low-energy (40 keV) and high-energy (100 keV) levels. A higher λHU value indicates a greater capacity for iodine uptake within the tumor. Effective-Z was computed using spectral CT material decomposition technology. It represents the mean atomic number value when the tissue within the ROI is equivalent to a single element. This parameter quantifies differences in the elemental composition of tumor tissue. Therefore, both λHU and Effective-Z were included in our analysis.

**Figure 1:**
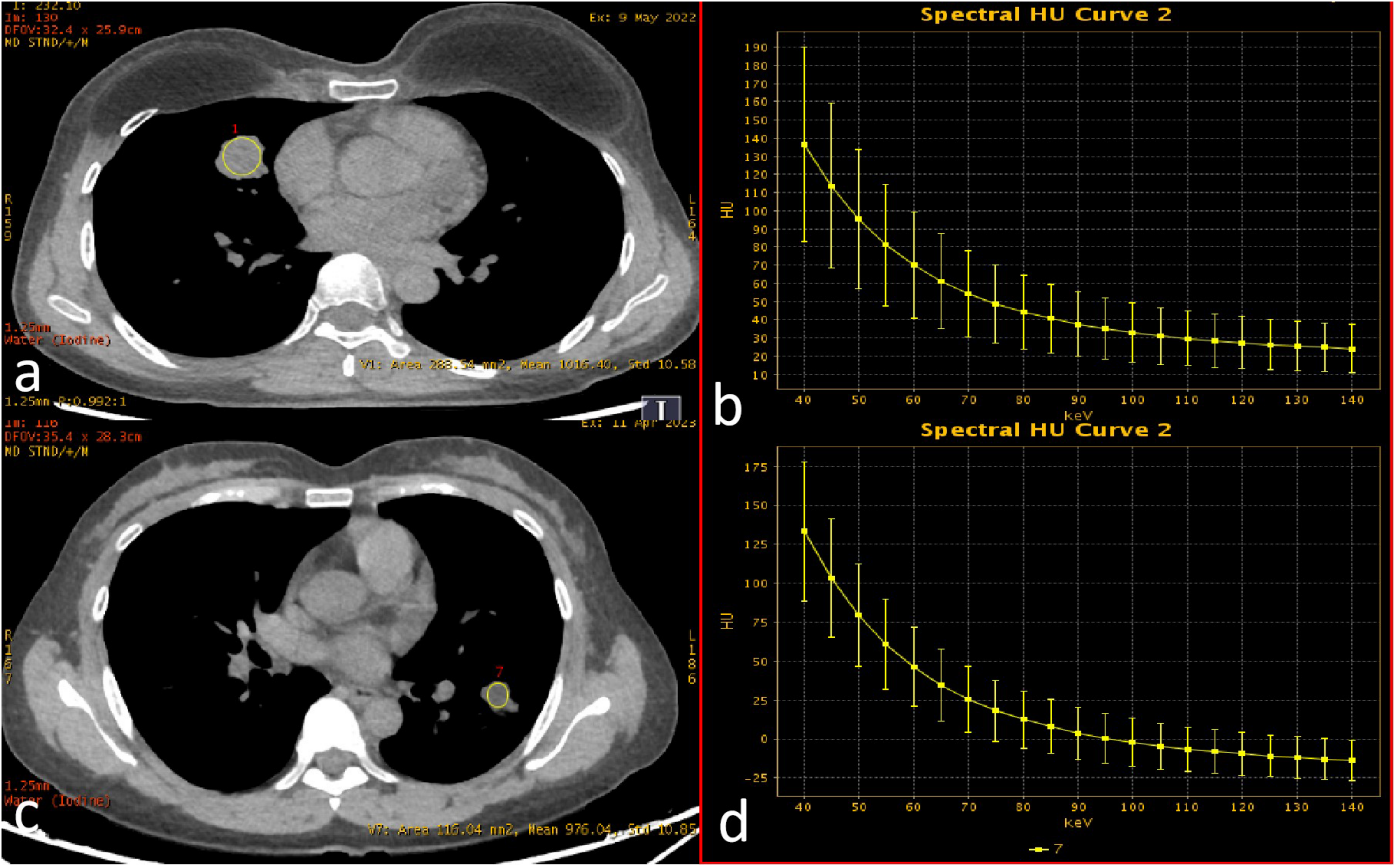
Spectral enhancement image with ROI delineation and spectral curves. ROIs were placed at the largest cross-sectional dimension of the tumor, encompassing the majority of solid components within the lesion. **(a, b)** EGFR wild-type (female, 45 years old; λHU = 1.59); **(c, d)** EGFR mutation-positive (female, 50 years old; λHU = 2.21).

Lesion delineation was independently performed by two radiologists (one attending radiologist and one resident radiologist), both blinded to patients’ clinical information and EGFR mutation status. The consistency of the observers’ measurement results was analyzed using the ICC. Final results were derived from the average measurements of both radiologists. In cases of significant interobserver discrepancy, a third senior radiologist with subspecialty expertise in thoracic imaging arbitrated the final determination. Representative images are shown in **Figure 1**.

### 2.4 AI parameter extraction

**Figure 2:**
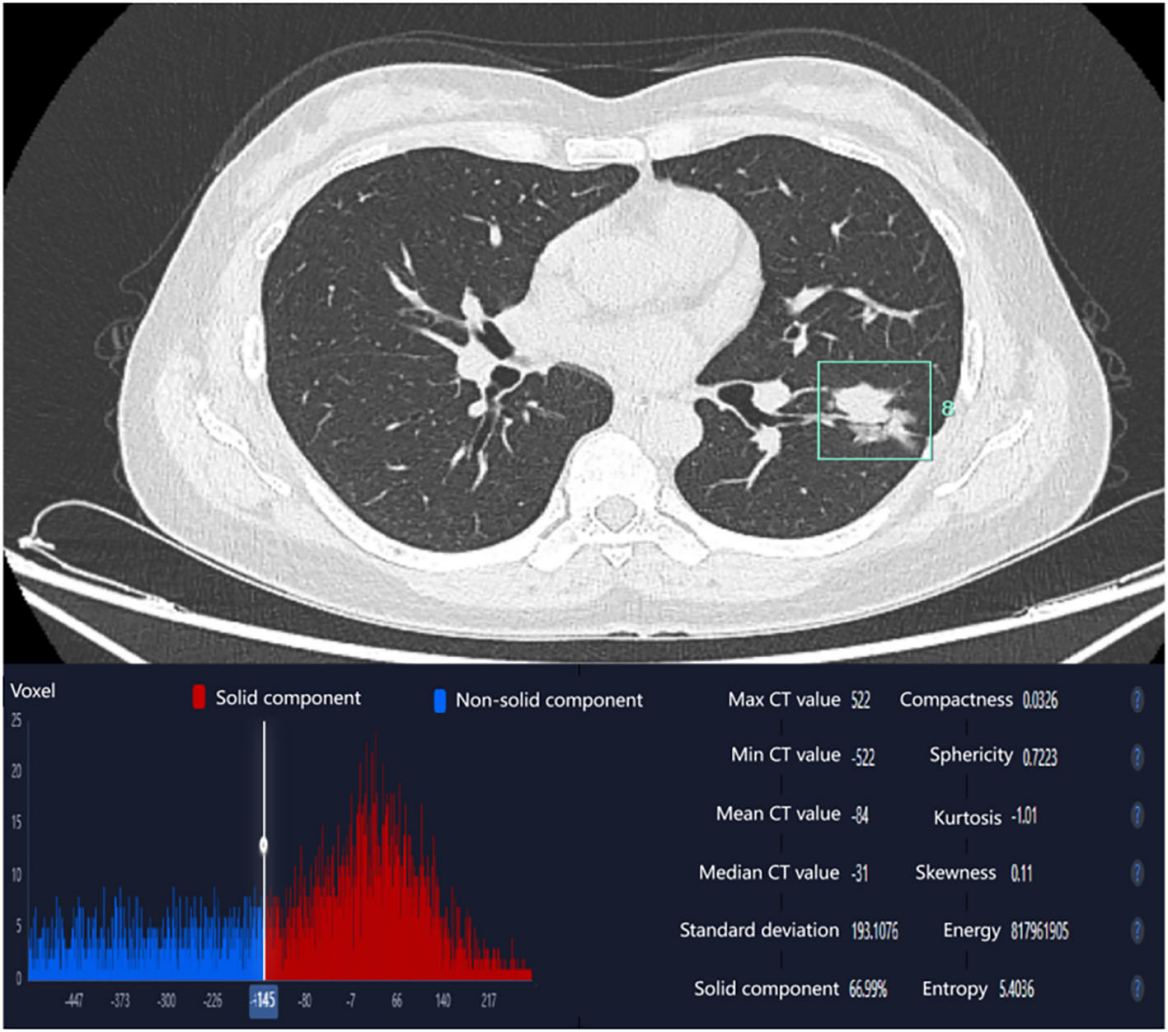
AI-derived CT histogram parameters. The solid component percentage was defined as the total number of voxels with CT attenuation ≥ -145 Hounsfield Units (representing the solid component) divided by the total number of voxels encompassing the entire tumor.

Non-contrast CT images (1.25 mm slice thickness, lung window: width 1200~1500 HU, level -600~-800 HU) were processed using an AI-powered pulmonary nodule analysis system (Infervision V4.0, Beijing, China). The system employs a pre-trained convolutional neural network to extract quantitative features from lesions in routine pulmonary CT examinations, with prior training and validation performed on CT datasets encompassing diverse acquisition parameters. The system automatically segmented tumors and generated 18 quantitative biomarkers: Three-dimensional (3D) radiomic features(including long-axis diameter, short-axis diameter, surface area, 3D long-axis diameter, maximum cross-sectional area, volume), CT attenuation histogram features(including solid component percentage, mean CT value, median CT value, CT value standard deviation, maximum CT values, minimum CT values, kurtosis, skewness, energy, and entropy)and morphological characteristics(including compactness, sphericity). Three-dimensional (3D) radiomic features enable a more comprehensive quantification of tumor burden. CT attenuation histogram features reflect intratumoral density variations, potentially indicative of pathological alterations such as necrosis, hemorrhage, or cellular proliferation. While morphological characteristics capture shape complexity, which may correlate with aggressive growth patterns and spatial heterogeneity. The AI-based pulmonary nodule diagnostic system leverages a deep learning algorithm to perform automatic segmentation of nodule boundaries and identification of typical radiological signs. For component analysis, the algorithm applies a threshold of -145 HU to differentiate solid from non-solid components within the nodule. It then calculates the number of voxels corresponding to each CT attenuation value, storing this data in a histogram. Subsequently, volumetric and other three-dimensional metrics are computed based on voxel counting (**Figure 2**) (17–19). Two board-certified radiologists independently validated AI-derived tumor segmentation accuracy through consensus review, ensuring spatial correspondence between imaging findings and postoperative histopathology.

### 2.5 EGFR mutation analysis

Pretreatment tumor specimens were obtained via surgical resection or needle biopsy. Formalin-fixed paraffin-embedded (FFPE) tumor tissue sections were subjected to microdissection to ensure a tumor cell content of ≥20%. EGFR mutations were detected using AmoyDx diagnostic kits(Xiamen, China), which employs the amplification refractory mutation system (ARMS) combined with fluorescent polymerase chain reaction (PCR) to identify EGFR mutations in tumor-derived DNA. This assay detects multiple EGFR mutation types, including exon 19 deletions (19-del), L858R, T790M, exon 20 insertions (20-Ins), G719X, S768I, and L861Q. All testing procedures were performed in strict accordance with the manufacturer’s protocols.

### 2.6 Statistical analysis

Statistical analysis was performed using IBM SPSS 23.0 software and R language (version 4.0.5). In univariate analysis, the normality of continuous variables was assessed using the Shapiro-Wilk test. Variables conforming to normal distribution were analyzed by independent samples t-test and expressed as mean ± standard deviation, while non-normally distributed variables were analyzed using the Mann-Whitney U test and presented as median (P25, P75). Categorical variables were compared using Pearson’s chi-square test or Fisher’s exact test. Variables demonstrating statistical significance in univariate analysis were subsequently incorporated into multivariate analysis. A binary logistic regression model with enter method was employed to identify independent influencing factors and construct a predictive model for EGFR mutations. The model’s discriminatory power, calibration, and clinical utility were assessed using Receiver Operating Characteristic (ROC) curve analysis, the Hosmer-Lemeshow goodness-of-fit test, and Decision Curve Analysis (DCA), respectively. For the comparison of inter-observer consistency of continuous variables, the intraclass correlation coefficient (ICC) is used. An ICC greater than 0.75 indicates good consistency. A *p*-value <0.05 was considered statistically significant.

## 3 Results

### 3.1 Clinical characteristics

A total of 150 lung adenocarcinoma patients were enrolled based on the inclusion and exclusion criteria, comprising 89 EGFR-mutant cases and 61 wild-type cases, with 72 males and 78 females (mean age 60.02 ± 9.15 years). Comparative analysis between the EGFR-mutant and wild-type groups revealed no statistically significant differences in age (*P* = 0.092), family history of lung cancer (*P* = 1.000), or previous cancer history (*P* = 0.202). However, statistically significant differences were observed in gender distribution (*P* = 0.013) and smoking history (*P* = 0.001) between the two groups (**Table 1**).

**Table 1:** Univariable logistic regression analysis of demographic characteristics.

Clinical characteristics	Group	Univariate logistic regression analysis			
		β-value	OR	95% CI	*P-value*
Age		-0.032	0.969	0.934-1.005	0.093
Gender	Male	Reference			0.011
	Female	0.867	2.379	1.221-4.634	
Smoking history	No	Reference			0.001
	Yes	-1.274	0.280	0.136-0.575	
Family history of lung cancer	No	Reference			0.975
	Yes	0.029	1.029	0.167-6.349	
prior malignancies	No	Reference			0.186
	Yes	1.069	2.914	0.597-14.222	

### 3.2 Comparison of the consistency of measurement results among observers

The measurement results of λHU, IC, WC, Effective-Z, and CT values at 70 keV by the two radiologists are shown in **Table 2**. The consistency of the data was good (ICC > 0.90).

**Table 2:** Measurement results and consistency between two observers.

Spectral CT parameters	Observer1	Observer2	ICC-value	P-value
λ_HU_	2.41 (1.92, 2.99)	2.37 (1.91, 2.89)	0.951 (95%CI:0.931-0.965)	<0.001
IC (mg/cm3)	20.02 (16.21, 25.34)	19.98 (16.18, 24.98)	0.948 (95%CI:0.928-0.963)	<0.001
WC (mg/cm3)	1008.29 (989.83, 1024.89)	1014.84 (991.69, 1025.18)	0.943 (95%CI:0.921-0.959)	<0.001
Effective-Z	8.81 (8.55, 9.06)	8.79 (8.52, 9.01)	0.938 (95%CI:0.913-0.956)	<0.001
70keV (HU)	62.13 (51.13, 73.67)	61.39 (48.96, 72.08)	0.946 (95%CI:0.924-0.961)	<0.001

### 3.3 Spectral parameter comparison

The EGFR-mutant group demonstrated significantly higher λ_HU_ values compared to the wild-type group (*P* = 0.049). However, no statistically significant differences were observed between the two groups in iodine concentration (*P* = 0.130), water concentration (*P* = 0.219), Effective-Z (*P* = 0.096), or 70 keV monochromatic imaging parameters (*P* = 0.650) (**Table 3**).

**Table 3:** Comparison of intergroup differences in spectral parameters.

Spectral CT parameters	EGFR mutant group (n=89)	EGFR wild-type group (n=61)	*Z*	*P-value*
λ_HU_	2.51 (1.98, 3.02)	2.19 (1.78, 2.90)	1.965	0.049
IC (mg/cm3)	21.21 (16.74, 25.47)	18.88 (15.44, 24.54)	1.513	0.130
WC (mg/cm3)	1009.35 (989.75, 1021.15)	1016.04 (993.04, 1024.87)	1.230	0.219
Effective-Z	8.83 (8.61, 9.10)	8.72 (8.51, 9.03)	1.666	0.096
70keV (HU)	61.38 (51.94, 73.75)	61.20 (48.86, 71.46)	0.453	0.650

### 3.4 AI-derived parameter comparison

Review by two physicians confirmed accurate spatial correspondence between the AI-identified tumor location and the postoperative histopathology findings. The EGFR-mutant group exhibited a significantly larger tumor surface area compared to the wild-type group (*P* = 0.043). However, no statistically significant differences were observed between the two groups in mean CT value (*P* = 0.830), median CT value (*P* = 0.789), solid component proportion (*P* = 0.995), maximum CT value (*P* = 0.431), minimum CT value (*P* = 0.895), CT value standard deviation (*P* = 0.477), longest diameter (*P* = 0.287), shortest diameter (*P* = 0.364), 3D longest diameter (*P* = 0.478), maximum cross-sectional area (*P* = 0.370), volume (*P* = 0.508), compactness (*P* = 0.056), sphericity (*P* = 0.055), kurtosis (*P* = 0.382), skewness (*P* = 0.576), energy (*P* = 0.243), or entropy (*P* = 0.356) (**Table 4**).

**Table 4:** Comparison of intergroup differences in AI parameters.

AI quantitative parameters	EGFR mutant group(n=89)	EGFR wild-type group(n=61)	*Z*	*P-value*
Long-axis diameter(cm)	3.07 (2.23,4.39)	2.62 (1.89,4.54)	1.064	0.287
Short-axis diameter(cm)	2.27 (1.65,3.31)	2.11 (1.35,3.24)	0.909	0.364
3D long-axis diameter(cm)	3.69 (2.66,5.21)	3.40 (2.38,5.39)	0.710	0.478
Maximum cross-sectional area(cm2)	5.03 (2.91,10.96)	4.15 (2.12,10.59)	0.897	0.370
Surface area(cm2)	28.57 (15.55,60.59)	18.63 (9.96,54.35)	2.026	0.043*
Volume (mm3)	7251.68 (2396.02,18434.39)	5384.57 (1585.68,19721.51)	0.662	0.508
Solid component percentage (%)	91.72 (57.79,100.00)	88.34 (64.57,100.00)	0.006	0.995
Mean CT value(HU)	33.00 (-36.00,61.00)	23.00 (-33.00,63.00)	0.214	0.830
Median CT value(HU)	36.00 (-3.00,55.50)	36.00 (-8.50,54.00)	0.268	0.789
CT value standard deviation	99.23 (77.83,143.68)	93.48 (69.40,139.35)	0.712	0.477
Maximum CT values(HU)	554.00 (436.00,682.00)	524.00 (415.50,651.50)	0.788	0.431
Minimum CT values(HU)	-213.00 (-372.00,-67.50)	-250.00 (-374.50,-53.00)	0.132	0.895
Compactness	0.0281 (0.0251,0.0326)	0.0312 (0.0255,0.0343)	1.913	0.056
Sphericity	0.6549 (0.6065,0.7230)	0.7026 (0.6134,0.7467)	1.917	0.055
Kurtosis	-0.87 (-1.01,-0.60)	-0.81 (-1.04,-0.56)	0.874	0.382
Skewness	0.17 (0.10,0.32)	0.18 (0.06,0.29)	0.559	0.576
Energy(×10^8^)	6.42 (3.57,15.33)	5.03 (2.14,12.97)	1.167	0.243
Entropy	4.78 (4.42,5.20)	4.60 (4.31,5.08)	0.922	0.356

### 3.5 Multivariate analysis

Variables demonstrating statistical significance in univariate analysis, including sex, smoking history, λHU, and tumor surface area, were subsequently subjected to multicollinearity evaluation. Linear regression confirmed variance inflation factors (VIF) of 1.999, 1.999, 1.336, and 1.319 for these variables, respectively, demonstrating the absence of significant collinearity or multicollinearity (VIF<2 threshold), thus warranting their inclusion in the multivariate regression model. The analysis identified smoking history (*P* = 0.012), λHU (*P* = 0.015), and tumor surface area (*P* = 0.029) as independent predictors of EGFR mutations, retaining statistical significance in the final model (**Table 5**).

**Table 5:** Results of multivariate regression analysis.

Variables		OR	95%CI	*P-value*
Gender	Male	Reference	Reference	0.879
	Female	0.926	0.344-2.495	
Smoking history	No	Reference	Reference	0.012
	Yes	0.256	0.088-0.744	
λ_HU_		1.972	1.144-3.399	0.015
Surface area		1.010	1.001-1.019	0.029

### 3.6 Construction and evaluation of the EGFR mutation prediction model

**Figure 3:**
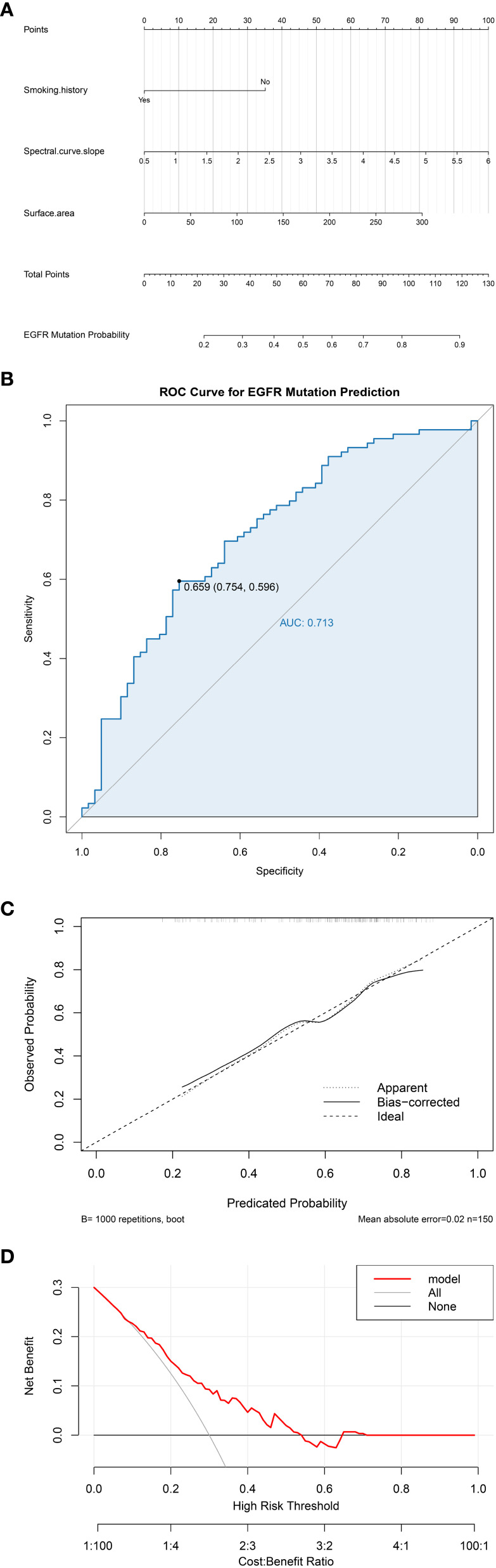
**(a)** Nomogram of the EGFR mutation prediction model; **(b)** Receiver operating characteristic (ROC) curve; **(c)** Calibration curve; **(d)** Decision curve analysis (DCA).

Independent risk factors identified through multivariate regression analysis (including smoking history, λHU, and tumor surface area) were integrated to develop a predictive model, with a nomogram constructed to visualize the results. The prediction model demonstrated strong discriminatory ability, as evidenced by an area under the ROC curve (AUC) of 0.713 (95% CI: 0.628–0.797). The model demonstrated a sensitivity of 0.600 and a specificity of 0.754. The Hosmer-Lemeshow goodness-of-fit test yielded χ² = 2.295 (P = 0.971), indicating no statistically significant difference between the model-predicted values and actual observed values. Furthermore, the calibration curve indicated favorable goodness-of-fit, and decision curve analysis revealed substantial clinical net benefit for patients when the threshold probability ranged between 6% and 54% (**Figure 3**).

## 4 Discussion

EGFR mutations represent the most prevalent genetic alterations in lung cancer among Asian populations, with an overall mutation rate ranging from 20% to 76% (2, 20).The prevalence of EGFR mutations is significantly increased in specific subpopulations, particularly among females (female *vs*. male: 43.7% *vs*. 24.0%; OR 2.7, 95% CI 2.5-2.9) and never-smokers (never-smokers *vs*. former/current smokers: 49.3% *vs*. 21.5%; OR 3.7, 95% CI 3.4-4.0), compared to other demographic groups (2, 3, 20). Studies have demonstrated a markedly elevated EGFR mutation rate in never-smokers and light smokers (21). This phenomenon may be attributed to the mechanisms through which tobacco smoking influences EGFR pathway activation, including oxidative stress induction and alterations in the cellular microenvironment, which subsequently modify EGFR functionality and activity (22, 23). In the present study, female gender and absence of smoking history were significantly more prevalent in the EGFR mutation group, with multivariate analysis identifying smoking history as an independent predictive factor for EGFR mutations. These findings align consistently with established research outcomes.

The EGFR signaling cascade governs cellular differentiation and division, playing a regulatory role in tumorigenesis and progression. Upon ligand binding, EGFR activation induces receptor dimerization and tyrosine autophosphorylation, thereby promoting vascular endothelial growth, tumor neovascularization, cellular proliferation, and enhanced tumor invasiveness (24). Consequently, administration of iodinated contrast agents reveals significant enhancement disparities between tumor vasculature and normal tissues (25). Clarifying EGFR mutation status provides crucial guidance for initiating and selecting therapeutic strategies in lung adenocarcinoma (26). Spectral CT leverages atomic number-dependent variations in X-ray attenuation coefficients to precisely analyze differences in material composition. The spectral curve illustrates continuous dynamic variations in material attenuation across different X-ray energy levels. A steeper spectral curve slope indicates greater dynamic attenuation changes, reflecting higher tumor enhancement intensity and vascular supply. Quantified by measuring CT value differences at two distinct monochromatic energy levels, this slope critically informs tumor composition analysis, enabling early diagnosis and therapeutic decision-making. Although iodine concentration and effective atomic number may partially reflect tumor vascularity, the current study found no statistically significant associations for these parameters. In contrast, the spectral curve slope demonstrated superior discriminatory capacity, with the EGFR mutation group exhibiting significantly steeper slopes than the EGFR wild-type group. We speculate that this may be attributed to the fact that the spectral curve slope reflects the dynamic attenuation changes in CT values across different monochromatic energy levels and is highly sensitive to detecting differences in the internal composition of lesions. Particularly in scenarios where precise monitoring of internal lesion changes is required, it provides more physical parameters than single iodine concentration or effective atomic number alone, thereby aiding in the comprehensive analysis of lesion characteristics from multiple perspectives. Multivariate analysis confirmed the spectral curve slope as an independent predictive factor for EGFR mutations, retaining superior predictive performance after adjusting for confounders.

EGFR promotes vascular endothelial cell proliferation, endowing tumor tissues with abundant blood supply and facilitating rapid tumor cell proliferation. Consequently, tumor burden correlates closely with tumor size, where larger dimensions typically indicate higher invasiveness. However, conflicting evidence from several studies (27–30) suggests that EGFR-mutant lung adenocarcinomas may exhibit smaller diameters compared to wild-type counterparts. This raises uncertainty regarding the reliability of tumor diameter as a predictor of EGFR mutation status. A critical limitation of conventional size measurement lies in its exclusive focus on the maximum cross-sectional diameter, neglecting multidimensional tumor characteristics. To address this, the present study employed an artificial intelligence (AI)-assisted diagnostic system to automatically identify tumors and perform integrated classification. This system generates multiple AI-quantified parameters, capturing not only one-dimensional (long-axis and short-axis diameters) and two-dimensional (area) metrics but also three-dimensional volumetric data, thereby overcoming the limitations of manual visual assessment. In univariate analysis, tumor surface area was significantly greater in the EGFR mutation group than in the wild-type group (P = 0.043), while other AI-derived parameters showed no statistical significance. Multivariate analysis further confirmed tumor surface area as an independent predictive factor for EGFR mutations. From a histopathological perspective, the EGFR gene mediates vascular endothelial cell growth and promotes neovascularization, thereby facilitating rapid aggressive tumor progression. During this process, tumor cells within the lesion demonstrate asynchronous growth rates in different directions, leading to varying degrees of fibrotic contraction and progressive infiltration into surrounding tissues. Consequently, highly aggressive tumors tend to develop characteristic spiculation and lobulation on their surfaces (28, 31). When tumors exhibit prominent spiculation and lobulation, this morphological alteration significantly increases the overall tumor surface area.

Dual-energy CT provides comprehensive tissue composition information, with its quantitative parameters reflecting tumor microstructural characteristics including vascularization, cellular density, and histopathological components - features potentially associated with EGFR mutation status. AI-driven CT parameter analysis enables extraction of radiomic features imperceptible through conventional methods. Our predictive model integrating spectral CT parameters, AI-quantified imaging biomarkers, and clinical indicators demonstrated discriminative capability for EGFR-mutant versus wild-type lung adenocarcinoma, achieving an AUC of 0.713 (95% CI: 0.628-0.797). Our findings align with and extend previous research on non-invasive EGFR mutation prediction using medical imaging. While CT radiomics has demonstrated substantial predictive capability in prior studies (32, 33), our work advances this field by incorporating functional insights from spectral CT parameters that quantify iodine uptake and tumor vascularity beyond conventional CT. Although PET/CT radiomics has shown excellent performance as reported by Deng et al. (34), our spectral CT approach provides complementary functional information within standard CT protocols. This multimodal integration strategy enables more comprehensive tumor characterization than single-modality approaches.

Liquid biopsy demonstrates a sensitivity of 35%–80% and specificity of 96%–100% for detecting EGFR mutations in lung cancer (35, 36). However, it exhibits false-positive rates of 6%–16% and false-negative rates as high as 42%–52% (36, 37). Our integrated model achieved moderate discriminative accuracy (AUC: 0.713) but offers distinct advantages over liquid biopsy: it circumvents the latter’s prohibitive costs and high false-negative rates, enables real-time integration into routine CT workflows, and proves more feasible for patients with insufficient DNA samples. Traditional CT radiomics studies typically rely on single-modality CT data and often utilize highly selected cohorts (e.g., stage IV lung cancer) (38, 39). Innovatively, this study combines multiparametric spectral CT physics with 3D AI-quantified features within a broader clinical population (stages I–IV), potentially conferring superior model generalizability. AI-derived parameters are automatically extracted during routine CT interpretation without significantly prolonging image analysis time. Crucially, the model leverages pre-acquired spectral CT data, eliminating the need for additional scans or associated costs. This non-invasive approach reduces unnecessary biopsies, particularly offering a viable alternative for patients contraindicated for invasive procedures. For suspected lung adenocarcinoma cases undergoing spectral CT, the model provides real-time EGFR mutation probability scoring during initial radiological evaluation. This enables clinicians to prioritize molecular testing for high-probability cases, thereby reducing diagnostic delays and optimizing resource allocation through streamlined precision oncology workflows. Continuous application during CT surveillance allows dynamic monitoring of EGFR mutation probability evolution, particularly valuable for cases with inconclusive biopsy results or those developing resistance to initial therapies. Such longitudinal assessment facilitates proactive therapeutic planning, securing critical treatment windows for patients. It is imperative to acknowledge, however, the inherent limitations of spectral CT as an imaging-based surrogate for direct molecular profiling. Unlike liquid biopsy or tissue genotyping, which directly detect genetic alterations, spectral CT infers mutation status indirectly through tumor phenotypic characteristics. This fundamental distinction implies that imaging features may not fully capture the complex genetic landscape of tumors. In summary, spectral CT and liquid biopsy should be regarded as complementary rather than competing modalities. The former provides rich structural and functional context, whereas the latter offers direct genetic evidence. Future research exploring the synergistic combination of radiomic features from spectral CT with circulating biomarkers may pave the way for more robust and comprehensive non-invasive tumor genotyping.

Although our integrated model achieved statistical significance and demonstrated moderate discriminatory power (AUC: 0.713), we acknowledge that its diagnostic performance, particularly the sensitivity of 60.0%, may currently limit its standalone utility in clinical practice. This moderate performance could be attributed to several factors. Firstly, despite extracting a comprehensive set of 18 AI-derived quantitative features and 5 spectral CT parameters, only a limited number (tumor surface area and λHU) were ultimately selected as independent predictors in the final multivariate model. This suggests that while high-dimensional feature spaces are explored, the most robust signals for EGFR mutation prediction might be concentrated in a few key characteristics. Many radiomic features, such as texture parameters (e.g., entropy, energy) and histogram metrics (e.g., kurtosis, skewness), which potentially reflect intratumoral heterogeneity, did not show significant univariate associations in our cohort. This could be due to the inherent biological complexity of lung adenocarcinoma, where the imaging phenotype influenced by EGFR mutation may be subtle and confounded by other genetic and microenvironmental factors. Second, our model construction relied on conventional binary logistic regression. Although robust and interpretable, this linear modeling approach may not fully capture potential nonlinear relationships or complex higher-order interactions among the imaging and clinical features. Future studies with larger sample sizes could employ more advanced machine learning algorithms to better decipher these intricate patterns and potentially improve predictive accuracy. Therefore, in its current form, the model is best positioned as a valuable non-invasive screening tool for risk stratification. It can help triage patients with a high probability of mutation for prioritized invasive testing, thereby optimizing resource allocation, rather than serving as a definitive substitute for tissue-based genotyping.

This study has several limitations. Firstly, this single-center retrospective study has a limited sample size (n=150), which may affect the generalizability of our model. To address this, we are currently collaborating with two tertiary hospitals to initiate a prospective multi-center validation study. While our integrated model demonstrates potential for the noninvasive prediction of EGFR mutations, several technical and practical considerations warrant careful attention prior to its clinical application. As indicated by our findings, the predictive accuracy of spectral CT is contingent upon both scan quality and the precision of region-of-interest (ROI) delineation. Variations in patient factors, scanner calibration, and contrast administration protocols can influence the quantification of spectral parameters. Such variability may affect the reproducibility of measurements across different institutions and scanner models. Furthermore, although our study employed a dual-observer approach with good interobserver consistency (ICC > 0.90), ROI placement remains inherently operator-dependent. Manual delineation introduces subjectivity, particularly in heterogeneous lesions or those with ill-defined margins. To mitigate this, we utilized consensus review and third-party arbitration in cases of discrepancy. Therefore, although our model offers a promising non-invasive alternative to biopsy, its real-world applicability must be validated in multi-center settings with standardized imaging protocols to ensure generalizability and consistency. Secondly, this study included cases across all TNM stages without subgroup analysis based on radiological nodule type, limiting deeper insight into EGFR prediction in early-stage disease. Multiple studies (28, 31–43) indicate that ground-glass nodules (GGNs) exhibit higher EGFR mutation rates than solid or part-solid nodules in pulmonary lesions <3 cm. However, as tumor stage advances and invasiveness intensifies, the proportion of solid components progressively increases. Whether this progression correlates with alterations in EGFR status remains to be elucidated. We will address this question in our multicenter study by performing stratified analyses according to tumor size (e.g., ≤3 cm *vs*. >3 cm), solid component proportion (e.g., pure GGN, part-solid), and T stage to refine prediction accuracy for specific clinical subgroups. Therefore, follow-up studies will further select cases based on different T stages. Thirdly, due to the current limitations of spectral analysis software, lesion region-of-interest (ROI) delineation is constrained to single-plane measurements that maximize two-dimensional lesion coverage. However, spatial variations across orthogonal tumor planes remain uncaptured, potentially resulting in loss of critical heterogeneity information. Future studies will employ integrated spectral-ct and radiomics approaches to implement three-dimensional volumetric tumor segmentation, enabling comprehensive heterogeneity quantification and enhancing the biological interpretability of predictive models.

In conclusion, spectral CT-derived quantitative parameters, AI-generated metrics, and clinical data demonstrate potential utility in predicting EGFR mutation status. Compared to invasive molecular testing techniques such as biopsy, the integration of spectral CT and artificial intelligence offers a safe, cost-effective, and non-invasive approach to assess tumor molecular profiles, thereby facilitating personalized therapeutic decision-making for patients.

## Data Availability

The raw data supporting the conclusions of this article will be made available by the authors, without undue reservation.
